# A Novel Closed-Head Model of Mild Traumatic Brain Injury Caused by Primary Overpressure Blast to the Cranium Produces Sustained Emotional Deficits in Mice

**DOI:** 10.3389/fneur.2014.00002

**Published:** 2014-01-22

**Authors:** Scott A. Heldt, Andrea J. Elberger, Yunping Deng, Natalie H. Guley, Nobel Del Mar, Joshua Rogers, Gy Won Choi, Jessica Ferrell, Tonia S. Rex, Marcia G. Honig, Anton Reiner

**Affiliations:** ^1^Department of Anatomy and Neurobiology, The University of Tennessee Health Science Center, Memphis, TN, USA; ^2^Department of Ophthalmology, The University of Tennessee Health Science Center, Memphis, TN, USA; ^3^Department of Ophthalmology and Visual Sciences, Vanderbilt University, Nashville, TN, USA

**Keywords:** mild TBI, anxiety, fear, depression, mice

## Abstract

Emotional disorders are a common outcome from mild traumatic brain injury (TBI) in humans, but their pathophysiological basis is poorly understood. We have developed a mouse model of closed-head blast injury using an air pressure wave delivered to a small area on one side of the cranium, to create mild TBI. We found that 20-psi blasts in 3-month-old C57BL/6 male mice yielded no obvious behavioral or histological evidence of brain injury, while 25–40 psi blasts produced transient anxiety in an open field arena but little histological evidence of brain damage. By contrast, 50–60 psi blasts resulted in anxiety-like behavior in an open field arena that became more evident with time after blast. In additional behavioral tests conducted 2–8 weeks after blast, 50–60 psi mice also demonstrated increased acoustic startle, perseverance of learned fear, and enhanced contextual fear, as well as depression-like behavior and diminished prepulse inhibition. We found no evident cerebral pathology, but did observe scattered axonal degeneration in brain sections from 50 to 60 psi mice 3–8 weeks after blast. Thus, the TBI caused by single 50–60 psi blasts in mice exhibits the minimal neuronal loss coupled to “diffuse” axonal injury characteristic of human mild TBI. A reduction in the abundance of a subpopulation of excitatory projection neurons in basolateral amygdala enriched in Thy1 was, however, observed. The reported link of this neuronal population to fear suppression suggests their damage by mild TBI may contribute to the heightened anxiety and fearfulness observed after blast in our mice. Our overpressure air blast model of concussion in mice will enable further studies of the mechanisms underlying the diverse emotional deficits seen after mild TBI.

## Introduction

Mild traumatic brain injury (TBI) is an extremely common outcome in military combat, sports, and vehicular accidents (1–3). Mild TBI, which involves either brief or no loss of consciousness, manifests with little, or no brain damage that can be detected by Magnetic Resonance Imaging (MRI), but typically is associated with “diffuse” axonal injury that is evident by diffusion tensor imaging (DTI) (4). Despite the minimal brain damage, mild TBI commonly leads to persistent psychologically debilitating symptoms that can include irritability, anxiety, fearfulness, and depression (1–3, 5–7). In many regards, these symptoms resemble those of post-traumatic stress disorder (PTSD), and in many cases victims of mild TBI are diagnosed as suffering from PTSD (4).

The prevalence of emotional disorders following mild TBI in humans has, however, only recently been recognized. As a result, the understanding of their pathophysiology is limited. Abnormalities in amygdalar and prefrontal cortical function have generally been linked to anxiety disorder, and hippocampal pathology and generalized axonal abnormalities have been linked to depression (8–12). However, whether alterations of these types occur following brain concussion and account for the specific emotional and cognitive symptoms of mild TBI remains unknown. Moreover, the means by which brain tissue deformation from a shock wave, blow to the head, or sudden deceleration yields axonal injury and/or brain dysfunction in specific brain regions associated with emotional functions has been uncertain (13, 14). Although emotional disorders stemming from concussive events have been reported in animal models, many of these models involve open skull, contusive injury such as those created in controlled cortical impact or fluid percussion paradigms (15), which causes more overt brain damage than does mild TBI. Fewer studies have used a closed-skull injury to create mild TBI to examine the link between brain trauma and emotional deficits. These studies have typically used a closed-head controlled impact or a whole-animal blast to create mild TBI, and many of these have focused on symptoms only within the first few weeks after the concussive event or events (15–23).

We have developed a mouse model of closed-head primary blast injury using a high-pressure air blast delivered to a 7.5-mm diameter mid-cranium area on the left side of the head. This model simulates the temporal and physical dynamics of the forces causing shock wave mediated mild TBI, such as would occur from an explosion or a blow to the head. Because only a small region of the head is targeted in our model, this system allows us to create TBI, without the hypoxia that may result from lung damage when the entire animal is exposed to the blast (17). We have found that a single 25–40 psi blast in C57BL/6 mice yields transient hyperanxiety, while C57BL/6 mice subjected to a blast of >40 psi show depression, fear, and hyperanxiety starting by 2 weeks post-blast, and lasting at least 2 months. The anxiety and fear deficits appear to be associated with decreased numbers of Thy1-enriched neurons in the basolateral amygdala (BLA). Our model thus simulates mild TBI in humans and the associated emotional disorders, and provides clues to their pathophysiology. The present paper describes these findings.

## Materials and Methods

### Animals

Adult male C57BL/6 mice were used in these studies. Mice were either purchased from Jackson Laboratories (Bar Harbor, ME, USA), and/or taken from a colony maintained from C57BL/6 founders from JAX. Three-month-old male mice were subjected to TBI caused by varying blast intensities, ranging from 0 to 60 psi, and the outcome evaluated behaviorally and histologically. We also used mice conditionally expressing enhanced yellow fluorescent protein (EYFP) in Thy1-expressing telencephalic neurons of the *emx1* lineage to histologically evaluate the effects of blast on excitatory neurons of the cerebral cortex and amygdala. These mice were obtained by breeding floxed Thy1-EYFP reporter mice and *emx1*-Cre driver mice (each of which was created on C57BL/6 background). Founder mice were purchased from JAX and colonies of each were established and maintained at UTHSC. The Thy1 promoter is widely expressed in projection neurons throughout the nervous system, whereas the Emx1 gene is selectively expressed in pyramidal neurons of cerebral cortex and amygdala. Cre-driven recombination occurs in nearly 90% of pyramidal cortical neurons. Thus, in the progeny of a cross between a floxed Thy1-EYFP reporter mouse and an Emx1-Cre driver mouse, nearly all excitatory cortical and many amygdalar neurons express EYFP (9, 24, 25).

Prior to blast exposure, mice were anesthetized with Avertin (400 mg/kg body weight), the fur on the parieto-temporal region of the left side of the head shaved, and the mouse secured in a specially designed mouse holder as described in the following section. Mice received 35 mg/ml Tylenol in their drinking water for 24 h after blast exposure. All animal studies were performed in accordance with a protocol approved by the Institutional Animal Care and Use Committee of the University of Tennessee Health Science Center, and complied with the National Institutes of Health and Society for Neuroscience guidelines.

### TBI methods

#### Blast device

**Figure 1:**
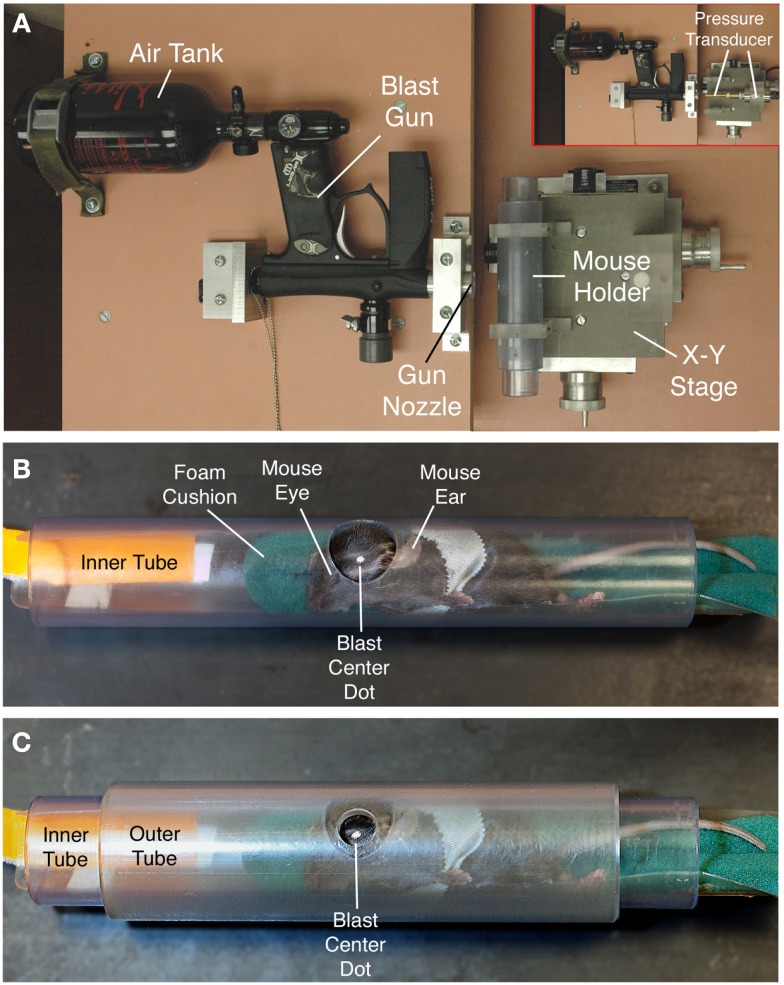
**The overpressure air blast in our model is delivered by a horizontally mounted modified paintball gun, as viewed from above in (A)**. The blast from the gun is regulated by adjustment of input from a pressurized air tank, and desired blast intensity experienced by the animal is calibrated using a pressure gauge to measure blast pressure output from the gun barrel at the position of the mouse head [inset in **(A)**]. To provide scale, the shorter, wider plastic tube perpendicular to the blast gun is 10.5-cm long. The inset shows the pressure transducer to the right, with its barrel passing through the region otherwise occupied by the mouse and mouse holder tubes. Anesthetized mice are secured within a foam rubber sleeve in a narrow PVC pipe with a 2.0-cm opening in one wall **(B)**, and slid into a just-wider PVC pipe with a 7.5-mm hole bored into its wall **(C)**. The two pipes are aligned so that the small outer hole exposes the temporal region of the mouse skull between ear and eye (the overlying fur is shaved, and a centering white spot marked on the skin). A foam rubber sleeve cushions the non-blast side of the mouse. The foam rubber is secured on a plastic sled with a tab by which the mouse holder can be pulled into the tube. The mouse is secured to the holder with several rounds of surgical tape.

An overpressure air blast of defined size, duration, and intensity was delivered by a small horizontally mounted air cannon system (26), consisting of a modified paintball gun (Invert Mini, Empire Paintball, Sewell, NJ, USA), pressurized air tank, and *x*-*y* table secured onto a medium-density fiberboard (Figure 1A). The original paintball gun barrel with a 13-mm aperture was replaced with a machined barrel with a 6.5-mm diameter aperture, to increase output pressure. The air blast pressures from the paintball gun are controlled by adjusting the output from a pressurized air tank, as monitored by a pressure gauge. The part of the mouse exposed to the blast is restricted to a 7.5-mm diameter mid-cranial area in the following manner (Figures 1B,C). Anesthetized mice are secured within a foam rubber sleeve in a 33-mm outer-diameter Plexiglas pipe (inner diameter – 26 mm) with the left side of the head positioned facing a 20-mm opening in the midpoint of one wall of the tube. This “inner” tube is slid into a 44-mm outer-diameter Plexiglas pipe (35-mm inner diameter) with a 7.5-mm hole bored into the midpoint of its wall. The inner tube is rotated in the outer tube so that the targeted head region is positioned in the center of the 7.5-mm hole in the outer tube. The outer tube is mounted perpendicular to the blast cannon with two Plexiglas holders that are mounted on an *x*–*y* table, which is itself mounted to the fiberboard (Velmex Inc., Bloomfield, NY, USA). Using the *x*–*y* controls, the outer tube opening is then aligned with the gun barrel opening, at a 10-mm distance from barrel tip (Figure 1A). This arrangement exposes the parieto-temporal region of the left side of the mouse cranium between ear and eye to the blast, while the rest of the mouse is shielded from the blast by the Plexiglas pipe. The foam rubber sleeve surrounding the mouse cushions the non-blast side of the mouse, to stabilize it and ensure that the blast does not produce head displacement that might cause an acceleration–deceleration injury to the brain.

#### Measurement of output pressures

In addition to the 7.5-mm hole that is positioned facing the gun barrel, the outer Plexiglas tube possesses an 11-mm hole on the opposite side, which allows insertion of the metal barrel of a model STJE Sensotec pressure transducer (Honeywell, Morristown, NJ, USA) to the position that the mouse head would occupy (26). The pressures detected by this pressure transducer are recorded and analyzed using Labview software (National Instruments, Austin, TX, USA). This makes it possible to precisely measure the air blast pressure at the position that the mouse head occupies during a blast session. We adjusted the gun pressure input from the compressed air tank to deliver pressures ranging from 0 to 60 psi at a distance of 10-mm from the gun barrel tip, which represents the position of the mouse cranium in relation to the blast gun barrel tip. This range includes pressures that were previously reported to produce TBI in rats exposed to whole-body blasts (27). Mice were weighed before blast and weekly thereafter.

### Behavioral studies

A series of behavioral tests was performed to characterize the impact of 0–60 psi blasts on fear, anxiety, and depression in the mice. The tests included open field behavior assessment, an acoustic startle test, a prepulse inhibition (PPI) test, a tail suspension depression test, and a fear acquisition and extinction test, with mice subjected to only one test on a given day. For one large set of mice, open field sessions were conducted the day before blast, the day after blast, 1 week after blast, and 2 weeks after blast. For some of these mice, behavioral tests of anxiety, fear, and depression were conducted between 6 and 8 weeks after blast, in order of least aversive to most aversive tests: (1) startle; (2) PPI; (3) tail suspension depression; and (4) fear acquisition and retention. A separate set of mice was used for examining tail suspension depression, and fear acquisition and retention at 2–3 weeks after blast without prior open field testing. Repeated Measures ANOVA with *post hoc* comparisons was used to evaluate the results statistically. ANOVA results are shown in the following form: [*F*(degrees of freedom, error) = *F* score; *p* = probability]. Statistical results for *post hoc* comparisons are shown in the following form: [*t*(degrees of freedom) = *t* score; *p* = probability].

#### Open field

We conducted automated 30-min assessments of open field behavior, using a Noldus EthoVision video tracking system to record and digitize mouse movements (Noldus Information Technology, The Netherlands), and the SEE software of Drai and Golani (28) to extract specific motor parameters from the locomotor record (29, 30). The SEE software uses algorithms to dichotomize mouse movements into progression segments of active locomotion and lingering episodes of little or no locomotion, with a speed threshold used to distinguish the two movement types. The SEE software further separates movement episodes and lingering episodes into those near the wall and those in the arena center (with center defined as more than 15-cm from the wall). The SEE software allows rapid characterization of numerous endpoints related to activity, locomotion, and anxiety, and has revealed behavioral differences among mouse strains (28, 31–34). The open field arena is 200-cm in diameter with a non-porous gray floor and a 50-cm high gray wall. Because of its particular relevance to anxiety disorder after blast, we present results here on the proportion of the open field session the mice spent lingering in the center of the arena.

#### Acoustic startle

Human victims of mild TBI and/or PTSD are hyper-responsive to aversive stimuli (1–3, 7, 35–37), which can be assessed in mice by the magnitude of the auditory startle responses to a series of randomly presented, abrupt loud tones. Tests were performed in 5.5-cm diameter–13 cm long Plexiglas cylinders, in a sound-attenuated chamber (SR-LAB, SDI, San Diego, CA, USA), with computer-controlled stimulus presentation and data acquisition, and a 63-dB white noise background. Mouse movements were detected by a piezoelectric accelerometer mounted under the cylinder floor. Startle amplitude is defined as the peak response during the first 100 ms after auditory startle stimulus onset. After 5-min for the mouse to acclimate in the cylinder, 10 startle stimuli at each of four different intensities (90, 100, 110, 120 dB) were presented, with an interstimulus interval (ISI) of 30-s between presentations. Startle stimuli were presented in blocks of four, with the four startle stimuli presented in random order within each block. A total of 10 trial blocks were presented.

#### Prepulse inhibition

Prepulse inhibition is a phenomenon in which a brief, sub-threshold stimulus (prepulse) inhibits the reaction of an animal to a subsequent strong startling stimulus (pulse). Defects in PPI have been observed after TBI and in PTSD (38–40), and may be related to a sensory or cognitive filtering deficit, for example the inability to filter out fear memories. Since PPI deficiency is a prominent endophenotype in schizophrenia (40), PPI defects in PTSD and after TBI may suggest the potential for neuropsychiatric disorders. For our PPI studies, the same chambers were used as for the acoustic startle tests. A speaker above the chamber provided acoustic pulses controlled by a computer, and a 63-dB background white noise. Each PPI test session consisted of six different trial types. Startle stimuli or “pulses” (115 dB, 50 ms) were presented alone or preceded by 20 ms auditory prepulses that were 2, 4, 8, 10, or 12 dB above the 63-dB background white noise (i.e., 65, 67, 71, 73, or 75 dB), with a fixed interval (100 ms) between the onsets of the prepulse and startle stimuli. The session began with a 5-min acclimation period followed by the six different stimulus presentation types presented in random order in blocks of six stimulus presentations over 9 blocks for a total of 45 trials. Intertrial intervals ranged from 20 to 40 s. Startle stimulus and prepulse intensities were selected based on prior studies of PPI in mice (41, 42). Startle inhibition was determined from the prepulse – pulse trials (100 ms prepulse – pulse interval) interspersed among the pulse-only trials, and data are presented here as % inhibition of the startle response obtained with the 115-dB tone alone.

#### Tail suspension depression test

Depression is a prominent symptom after TBI in humans (43). To assess depression-like behavior in mice, the tail suspension test was used (44). In this paradigm, mice suspended by their tail eventually stop attempting to escape and become immobile, with a depressive-like state indicated by a short latency to immobility and longer duration of immobility over the test period. For this test, each mouse was suspended above a solid surface by the use of vinyl adhesive tape applied to the tail so that its body dangled in the air, facing downward. Measures of immobility were recorded and analyzed over a 5-min period using a digital video camera interfaced with a computer and automated software (FreezeFrame, Coulbourn, Whitehall, PA, USA).

#### Fear acquisition and extinction tests

Human victims of mild TBI tend to show enhanced retention of fearful memories (11). We tested for increased fear retention in our mild TBI mice following fear conditioning using a Pavlovian fear-learning paradigm. The fear-conditioning chamber possesses clear polycarbonate walls and a stainless steel grid floor (MED Associates, Model ENV-008), and is fitted with a video camera interfaced with a computer. Foot shock stimuli and conditioned freezing responses are controlled, collected, and analyzed using automated software (FreezeFrame, Coulbourn, Whitehall, PA, USA). Five minutes after being placed in the fear-conditioning chamber, mice received five fear training trials, each consisting of a 30-s tone (12 kHz) co-terminating with a 0.250-s, 0.4-mA foot shock, with a variable intertrial interval with a mean of 2-min (range, 1–3 min). On each of the following 3 days, mice were given extinction sessions, each consisting of 15 tone alone test trials, with freezing again the measure of fear, again at a variable intertrial interval with a mean of 2-min. Contextual fear was assessed in a separate set of 0-, 30-, and 60-psi blast mice 3 weeks after blast. These mice received two conditioned stimulus (CS)-shock pairings, each consisting of a 20-s, 12-kHz tone co-terminating with a 2-s, 0.7-mA foot shock administered 2 and 3 min after being placed in the fear-conditioning chamber. The following day, contextual fear was assessed in the absence of the CS cue by examining freezing responses after mice were returned to the conditioning chamber. The contextual fear test duration was 10-min, and freezing was sampled over 20-s blocks. Contextual fear was indicated by the presence of freezing during the first 3-min period (the time during which they had received shock during training).

### Histological studies

After behavioral testing had been completed, animals were sacrificed by transcardial perfusion with fixative, and several types of histological analysis carried out to determine the effects of TBI on brain: (1) immunolabeling for NeuN, glial fibrillary acidic protein (GFAP), ionized calcium binding adaptor molecule 1 (IBA1), and SMI-32; (2) cresyl violet Nissl staining; (3) hematoxylin and eosin (H&E) staining; and (4) NeuroSilver staining. Additionally, mice conditionally expressing EYFP in Thy1-expressing telencephalic neurons of the *emx1* lineage were used to histologically evaluate the effects of blast on pyramidal neurons of the cerebral cortex and amygdala. Behavioral analysis was not carried out on the mice conditionally expressing EYFP. More detailed methods for these various histological studies are presented below.

#### Tissue fixation

Mice were deeply anesthetized (Avertin; 400 mg/kg), and perfused transcardially with 40 ml of 0.9% NaCl, followed by 200 ml of 4% paraformaldehyde, 0.1 M lysine-0.1 M sodium periodate in 0.1 M sodium phosphate buffer at pH 7.4 (PB). The brains were removed, stored overnight in the same fixative at 4°C, and then stored for at least 24 h in a 20% sucrose/10% glycerol solution at 4°C. The fixed brains were sectioned frozen on a sliding microtome in the transverse plane at 35 μm. Each brain was collected as 12 separate series in 0.1 M sodium phosphate buffer (pH 7.4) containing 0.02% sodium azide, and stored until processed for immunohistochemistry or NeuroSilver. A one in six series of brain sections from each mouse was mounted as it was sectioned, and subsequently stained for cresyl violet. An additional series was later mounted and stained for H&E.

#### Immunohistochemical studies

Immunohistochemical single-labeling using peroxidase– antiperoxidase (PAP) procedures as described previously (45) was employed to visualize a variety of neurochemical markers in TBI brains. Immunolabeling for NeuN was used to detect neuronal perikarya, using a mouse monoclonal anti-NeuN (MAB377, Millipore Corp., Billerica, MA, USA). The specificity and efficacy of the anti-NeuN antibody, which targets Fox-3, a new member of the Fox-1 gene splicing family (46), has been shown previously (47, 48). Astroglial upregulation of GFAP is a standard indicator of brain injury that we have employed in prior studies (49, 50). We performed immunolabeling to detect astroglial reaction to TBI with a mouse anti-GFAP antibody (AB6, Thermo Scientific, Fremont, CA, USA). We performed immunolabeling to detect microglial reaction to TBI with a rabbit anti-IBA1 antibody (019-19741, Wako, Richmond, VA, USA). The mouse monoclonal SMI-32 antibody (SMI-32R, Covance Inc., Princeton, NJ, USA) was used to evaluate cortical pathology after TBI. The SMI-32 antibody we used detects a non-phosphorylated epitope on neurofilament H (200 kDa), which is abundant in neuronal cell bodies and dendrites, especially those of cortical neurons (51). As the phosphorylation state of neurofilament H is sensitive to cortical neuron disease and injury (52, 53), the SMI-32 antibody can help reveal traumatic injury to cerebral cortex.

To carry out immunohistochemical labeling, sections were incubated for 24 h at room temperature in primary antibody diluted 1:2000–1:5000 with 0.8% Triton X-100/0.01% sodium azide/0.1 M PB (PBX). Sections were subsequently incubated in donkey anti-mouse IgG diluted 1:200 in PBX, followed by incubation in mouse PAP complex diluted 1:1000 in PBX without sodium azide, with each incubation at room temperature for 1 h. The sections were rinsed between secondary and PAP incubations in three 5-min washes of PB. Subsequent to the PAP incubation, the sections were rinsed with three to six 10-min washes in 0.1 M PB, and a peroxidase reaction using diaminobenzidine tetrahydrochloride (DAB) was carried out. Sections were incubated in 5 ml of 0.05 M imidazole/0.05 M cacodylate buffer (pH 7.2) containing 5 mg DAB for 10-min and then incubated for an additional 10-min after adding 20 μl of 3% hydrogen peroxide. Sections were then rinsed in distilled water, transferred to 0.1 M PB, mounted onto gelatin-coated slides, dried, dehydrated, and coverslipped with Permount^®^.

#### NeuroSilver

The NeuroSilver stain was used to detect degenerating axons (17). For silver staining of injured/degenerating axons and terminals, sections were processed with a commercially available modification of the Gallyas silver method (NeuroSilver kit II; FD Neurotechnologies, Ellicott City, MD, USA), following manufacturer instructions (8).

#### Thy1-EYFP mice

Thy1-EYFP mice were transcardially perfused with fixative and sectioned as described above. Sections were mounted onto gelatin-coated slides, dried, dehydrated, and coverslipped with ProLong^®^ antifade medium (Molecular Probes, Eugene, OR, USA). Sections were viewed and images captured using a Zeiss 710 confocal laser scanning microscope (CLSM). Labeled neuron counts were performed using NIH Image J (version 1.48b).

## Results

### Behavioral studies

#### Open field

**Figure 2:**
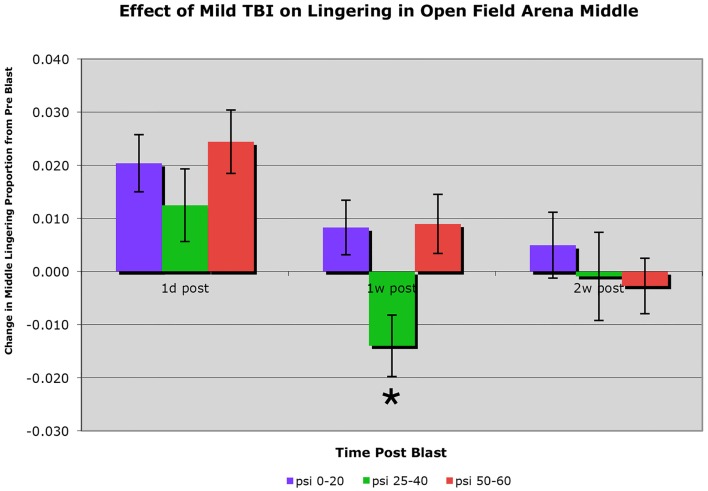
**Graph showing the effects of 50–60 psi blasts compared to 0–20 and 25–40 psi blasts on the proportion of a 30-min session that mice spent lingering in the center of an open field arena at 1 day, 1 week, and 2 weeks after blast**. Data are expressed as the change from pre-blast baseline for each post-blast time point. Note that lingering in the middle of the open field arena is significantly decreased (asterisk) in the 25–40-psi mice at 1 week but not 2 weeks, compared to the 0–20-psi mice. By contrast, lingering in the middle of the open field arena in the 50–60-psi mice is progressively diminished compared to 0–20 psi mice from 1 to 2 weeks after blast.

C57BL/6 mice subjected to a single blast of 40-psi or less showed no obvious motor deficits in open field, such as a decrease in speed or total distance traveled (i.e., activity). Mice with a 25–40-psi blast (*n* = 81) did, however, linger in the middle of the open field arena significantly less than mice with a 0–20 psi blast (*n* = 50) or a 50–60 psi blast (*n* = 76) at 1 week after blast (*p* < 0.01), the greater avoidance of the arena center by the 25–40-psi mice being indicative of increased anxiety (Figure 2). This difference among groups for middle lingering was not present 1 day after blast [*F*(2, 204) < 0.88; *p* > 0.05] or before blast (data not shown). By 2 weeks, the hyperanxiety following 25–40-psi blasts that had been evident at 1 week was diminished, with middle lingering no longer significantly less than for the 0–20-psi mice. The 50–60-psi mice (*n* = 76), by contrast, showed no indication of increased anxiety in open field at 1 week after blast, but did show mild deficits in speed and distance traveled. The 50–60-psi mice, however, appeared to show a trend over time toward hyperanxiety, lingering in the arena middle <0–20 psi mice at 2 weeks post-blast (Figure 2).

Because TBI-induced hyperanxiety appeared to increase in mice receiving 50–60 psi blasts by 2 weeks after blast, we performed additional tests of emotional distress on a subset of the same 50–60 psi mice (*n* = 24), compared to some of the same 0-psi sham mice (*n* = 31) and 30-psi mice (*n* = 7), at 6–8 weeks. These additional tests included startle, PPI, tail suspension depression, and fear conditioning and extinction. In a separate set of 0-, 30-, and 60-psi mice, we evaluated tail suspension depression at 2 weeks post-blast, and contextual fear after fear conditioning at 3 weeks after blast. The results are described below.

#### Acoustic startle

**Figure 3:**
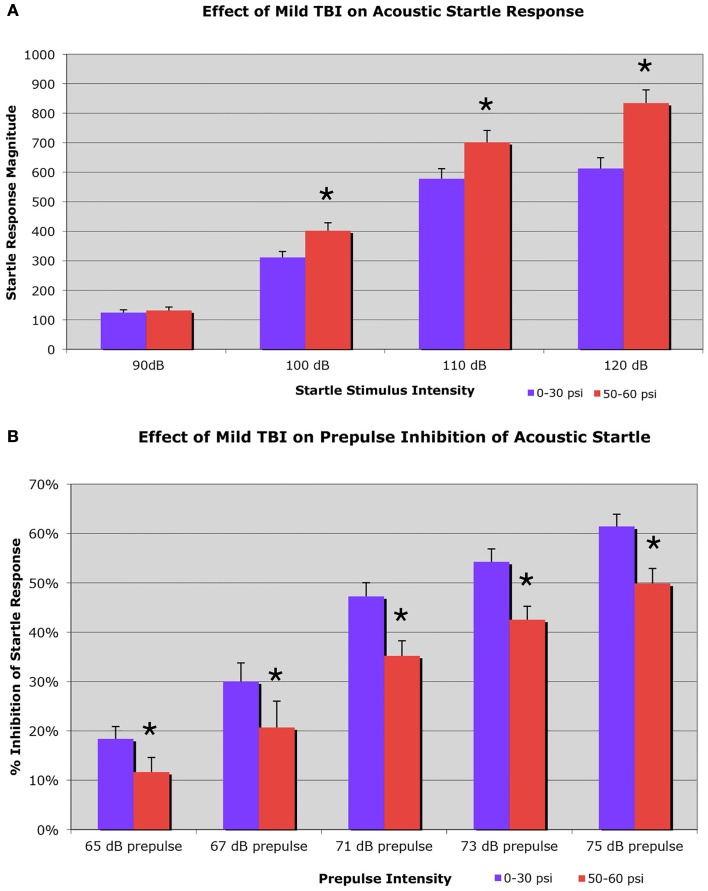
**Graphs showing: (A) the effects of 50–60 psi blasts compared to 0–30 psi blasts in mice on the startle response to a series of loud tones 6–8 weeks after blast; and (B) the effects of 50–60-psi blasts compared to 0–30 psi blasts in mice on prepulse inhibition 6–8 weeks after blast**. Note that startle is enhanced in 50–60-psi mice compared to 0–30 psi mice, and prepulse inhibition is markedly attenuated in the 50–60-psi mice compared to 0–30 psi mice. Asterisks indicate a significant difference between the 0–30 and 50–60 psi mice.

On the startle test, conducted 6–8 weeks after blast, we found an elevated startle response to a 100-dB (29.2% increase), a 110-dB (21.4% increase), and a 120-dB auditory tone (36.2% increase), in a sample of 37 mice with 50–60 psi blast compared to 54 mice with 0–30 psi blast (Figure 3A). Two-way ANOVA showed significant main effects of startle intensity [*F*(3, 267) = 301.21; *p* < 0.01] and group [*F*(1, 89) = 11.35; *p* < 0.01], as well as a significant stimulus intensity × group interaction [*F*(3, 267) = 7.99; *p* < 0.01]. Follow-up comparisons for individual startle stimuli indicated that 50–60 psi mice showed significantly higher responses than mice with 0–30 psi blast at startle intensities of 100, 110, and 120 dB [*t*s(89) > 2.35; *p*s < 0.05]. Mice receiving 50–60 psi blast were thus hyper-responsive to a startle stimulus, as is common in human victims of TBI.

#### Prepulse inhibition

Mice receiving 50–60 psi blast (*n* = 38) showed notably attenuated PPI 6–8 weeks after blast, compared to mice with 0–30 psi blast (*n* = 56), for a range of prepulse intensities (Figure 3B). For example, mice with 0–30 psi blast exhibited 61.4% inhibition of the startle response by the loudest prepulse (75 dB), while mice with 50–60 psi blast showed only 49.9% inhibition. Two-way ANOVA showed significant effects of prepulse intensity [*F*(4, 368) = 67.01; *p* < 0.01] and group [*F*(1, 92) = 9.53; *p* < 0.01], as well as a significant prepulse intensity × group interaction [*F*(4, 368) = 5.29; *p* < 0.01]. Follow-up comparisons showed 50–60 psi mice exhibited significantly lower PPI at all prepulse intensities (i.e., 65, 67, 71, 73, or 75 dB) [*t*s(92) > 2.01; *p*s < 0.05]. Thus, mild TBI caused by a single 50–60 psi blast diminished PPI. As diminished PPI is observed in schizophrenia, these results indicate that a 50–60 psi blast produces signs of a psychiatric disorder in our mice.

#### Tail suspension depression

**Figure 4:**
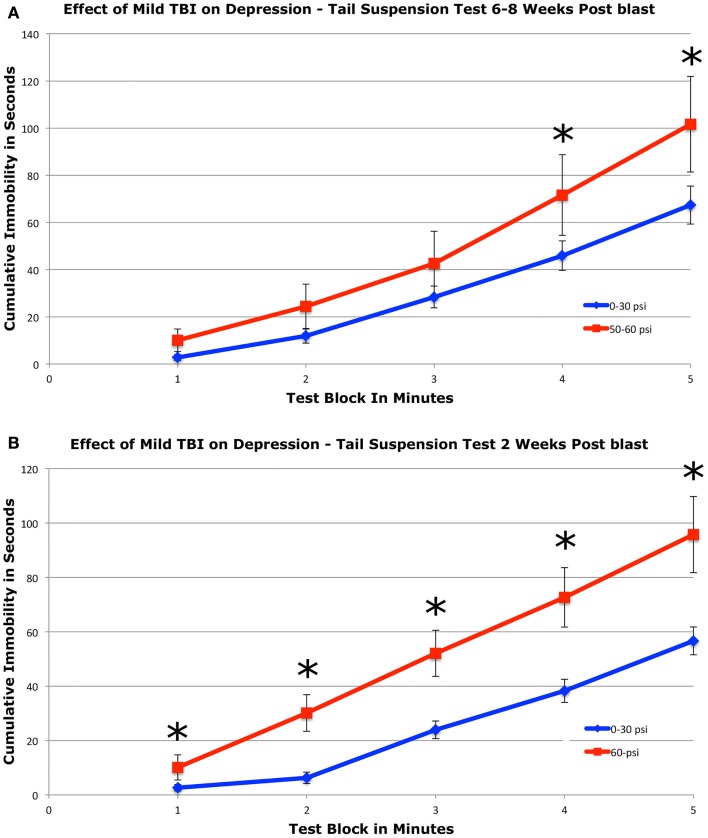
**Graphs showing the effects of 50–60 psi blasts compared to 0–30 psi blasts in mice on the tail suspension test of depression 6–8 weeks (A) and for 60-psi blasts compared to 0–30 psi blasts 2 weeks (B) after blast**. Data are presented as cumulative immobility per 1-min time block. Note that immobility, reflecting a depression-like behavior, is increased in the 50–60-psi mice compared to the 0–30-psi mice. Asterisks indicate a significant difference between the 0–30 and 50–60 psi mice.

To assess depression, we used a 5-min tail suspension test. Numerous studies have shown that depression in mice can be detected by an increase in immobility in this assay (44, 54, 55). We found that mice with 50–60 psi blast (*n* = 17) showed a significant 51.2% increase in immobility during the last 2-min of the test compared to mice with 0–30 psi blast (*n* = 24), 6–8 weeks after blast (Figure 4A). Group differences were evaluated statistically in 1-min blocks over the 5-min test. This analysis revealed significant main effects of group [*F*(1, 39) = 4.33; *p* < 0.05] and test time block [*F*(4, 156) = 23.70; *p* < 0.01], but no group × time interaction [*F*(4, 156) = 1.23; *p* > 0.05]. The 50–60-psi mice thus showed greater immobility than 0–30 psi mice, indicating a “depression-like” behavioral phenotype. Follow-up *t*-tests revealed significant differences between groups at minutes 4 and 5 of the tail suspension test [*t*s(39) > 2.03; *p*s < 0.05], but no differences during the first 3-min [*t*s(39) < 1.77; *p*s > 0.05]. Results are presented graphically as cumulative percent immobility for 0–30 psi compared to 50–60 psi mice (Figure 4A).

To determine if a 50–60-psi blast produces depression at earlier times after blast, we tested a separate set of mice at 2 weeks after blast (Figure 4B). Mice subjected to 60-psi (*n* = 13) blast showed increased immobility in the tail suspension test as compared to 0- and 30-psi mice (*n* = 23), the latter two of which did not differ from one another. A repeated-measure ANOVA indicated significant main effects of group [*F*(1, 34) = 11.06; *p* < 0.01] and test time block [*F*(4, 136) = 10.54; *p* < 0.01], but no group × time interaction [*F*(4, 136) = 1.76; *p* > 0.05]. Follow-up *t*-tests revealed significant differences between groups at minutes 1 and 2 of the tail suspension test [*t*s(34) ≥ 2.16; *p*s < 0.05], but no differences during the last 3-min [*t*s(34) ≤ 0.91; *p*s > 0.05]. Results are presented graphically as cumulative percent immobility for 0–30 psi compared to 60 psi mice (Figure 4B). Thus, mild TBI stemming from 50 to 60 psi single blasts increased depression in mice by 2 weeks after the concussive event, which persisted for at least 2 months.

#### Fear conditioning, retention, and extinction

**Figure 5:**
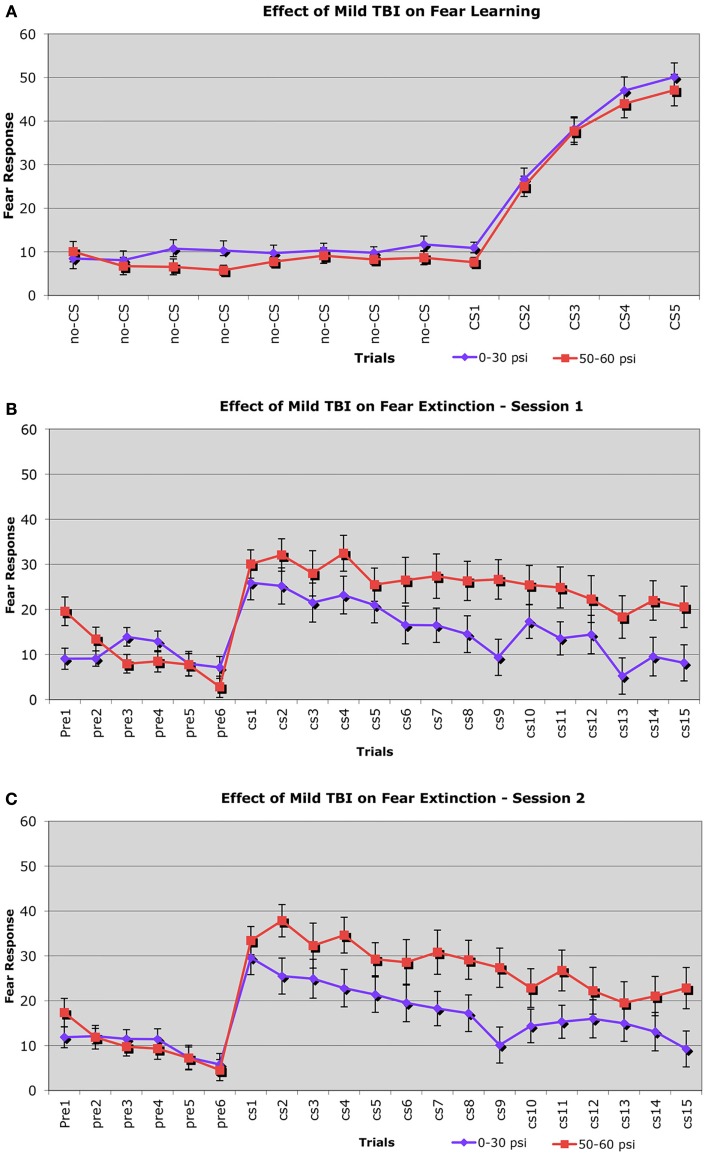
**Graphs showing: (A) the effects of 50–60 psi blasts compared to 0–30 psi blasts in mice on the acquisition of conditioned fear to an auditory cue signaling impending shock at 6–8 weeks after blast; (B) the effects of 50–60 psi blasts compared to 0–30 psi blasts in mice on extinction of conditioned fear to an auditory cue signaling impending shock, on the day after the conditioning shown in (A); and (C) the effects of 50–60 psi blasts compared to 0–30 psi blasts in mice on a second day of extinction of conditioned fear to an auditory cue signaling impending shock, 2 days after the conditioning shown in (A)**. Note that 50–60 psi mice do not differ from 0 to 30 psi mice in fear acquisition, but do show more fear retention and lesser fear extinction during the 2 days of extinction testing. Fear extinction session performance has been normalized for both groups to pre-fear conditioning baseline levels, to remove pervasive contextual effects.

Fear learning was assessed using a fear conditioning–fear extinction paradigm 6–8 weeks after blast. During the fear conditioning session, freezing scores in response to the CS (the auditory cue) increased similarly in both 50–60 psi mice (*n* = 38) and 0–30 psi mice (*n* = 56) from the first to the last paired CS – shock (unconditioned stimulus, US) presentation (Figure 5A). Thus, 50–60 psi blast did not alter the acquisition of a learned fear response to an auditory cue signaling impending foot shock. Accordingly, the ANOVA indicated a significant main effect of trial [*F*(4, 39) = 32.4, *p* < 0.01], but no group effect or group × trial interaction [*F*s < 1.0, *p*s > 0.05]. Nonetheless, mice with 50–60 psi blast did show significantly increased fear in response to the CS during the subsequent extinction tests (Figures 5B,C). During both the first and second extinction sessions (performed on successive days after fear acquisition), the 50–60-psi mice showed a greater fear response to the CS than did the 0–30-psi mice. Moreover, unlike the response in the 0–30-psi mice, the fear responses during the last few CS presentations did not decline to basal levels (i.e., the level prior to CS presentation). By the third extinction session, however, the responses of the 50–60-psi mice were indistinguishable from those observed in the 0–30-psi mice, with both groups showing similar fear responses to the CS over the 15 presentations, and fear responses in both groups declining to basal levels by the last CS presentation (data not shown). A two-way ANOVA with group and extinction session as independent variables revealed significant effects of session [*F*(2, 184) = 5.38, *p* < 0.01], and a group × test session interaction [*F*(2, 184) = 4.48, *p* < 0.02]. Thus, the 50–60-psi mice behaved significantly differently than the 0–30-psi mice during the extinction sessions. Independent *t*-tests for each extinction session revealed a nearly significant overall difference between the 0–30 and 50–60 psi groups for extinction session 1 [*t*(28) > 1.93; *p* = 0.056]. For extinction session 2, the independent *t*-tests indicated that the 50–60-psi mice showed significantly more freezing than the 0-psi mice [*t*(28) = 2.11; *p* < 0.05], indicating a greater retention of fear in the 50–60-psi mice during extinction session 2. Both groups displayed statistically similar levels of CS-elicited freezing during extinction session 3. Thus, our results in the mice trained with five fear conditioning trials, and tested with three daily 15 CS-only extinction trials show that mild TBI caused by 50–60 psi blast did not affect the initial learning but enhanced retention and slowed extinction of learned fear 6–8 weeks after the concussive event. However, with the additional training provided by the third extinction session, 50–60 psi mice showed extinction of the fear response to the CS tone.

#### Fear conditioning and contextual fear

**Figure 6:**
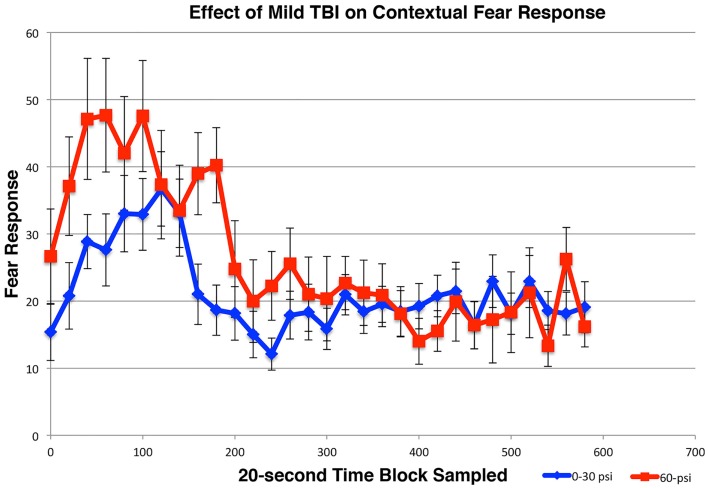
**Graph showing the effects of 60-psi blasts compared to 0–30 psi blasts on contextual fear in mice subsequent to acquisition of conditioned fear to an auditory cue signaling impending shock, 3 weeks after blast**. Freezing during successive 20-s time blocks over a 600-s test period is shown. Note that contextual fear is enhanced in 60-psi mice compared to 0–30 psi mice, during the first 2-min of the test.

For the first and second extinction sessions described above, the 50–60-psi mice showed a fear response upon entry into the conditioning chamber itself (Figures 5B,C), which was not evident in the 0–30-psi mice. Thus, the mice with mild TBI appeared to show a generalization of the fear to their environment after five fear-conditioning trials, which was not evident in the control mice. To further investigate the effects of mild TBI on fear learning and generalization, we tested fear acquisition and generalization 3 weeks after blast in a separate group of mice with 0-psi (*n* = 12), 30-psi (*n* = 12), or 60-psi (*n* = 12) blast, in this case after only two fear training trials. Again, we found no differences in fear acquisition among 0-, 30-, and 60-psi mice (*p* > 0.05). We then examined conditioned contextual fear in these mice, which in contrast to amygdala-dependent cue-specific fear learning is reportedly dependent on both the hippocampus and amygdala (56, 57). One day after training, the mice were returned to the training chamber, where conditioned freezing was assessed in the absence of CS or shock presentations. All groups showed a contextual fear response over the first 3-min in the training chamber, but the 60-psi mice showed a significantly greater contextual fear response than the 0- and 30-psi mice (Figure 6), as indicated by a significant main effect of group [*F*(1, 34) = 10.89; *p* < 0.01] and time [*F*(2, 68) = 3.96; *p* < 0.05]. The group × time interaction [*F*(2, 68) = 31.11; *p* > 0.05] was not significant. Thus, in addition to increased retention and lessened extinction of learned fear to a cue signaling shock, 50–60 psi mice showed greater fear to the training context as well.

### Histological analysis

**Figure 7:**
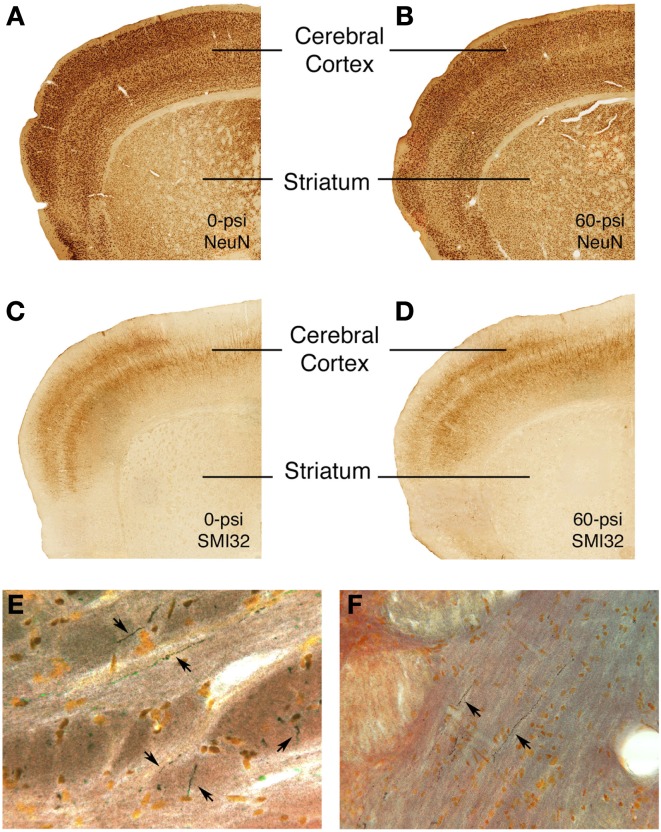
**Images showing the absence of neuronal damage but the presence of axonal injury after 50–60 psi blasts**. Images **(A–D)** present sections through the rostral cerebral cortex on the blasted side immunolabeled for NeuN (a pan-neuronal marker) **(A,B)** and SMI-32 (a marker of perikarya and dendrites of layer 3 and layer 5 cerebrocortical neurons) **(C,D)** from 0- or 60-psi mice, 3–4 weeks after blast. Note the absence of evident neuron loss or injury in cerebral cortex or striatum in the 60-psi mice. By contrast, degenerating axons as detected by NeuroSilver staining are seen above the pyramidal tract **(E)** in a mouse sacrificed 2 weeks after a 50-psi blast, and in the lateral lemniscus **(F)** in a mouse sacrificed 2 weeks after a 60-psi blast.

Sections through the brain area targeted by the blast from all mice tested behaviorally were stained with cresyl violet and H&E. We observed no obvious differences in the cerebral cortex of mice that received 50–60 psi blast as compared to those that received 25–40 psi blast or 0–20 psi blast. Thus, there appears to be no evident neuron loss following 20–60 psi blast. Similarly, neither immunolabeling for the pan-neuronal marker NeuN, nor immunolabeling for SMI-32, which is especially abundant in the cell bodies and dendrites of layer 3 and 5 cortical neurons, was attenuated in the cortical blast territory on the left side of the brain, even in those mice with 50–60 psi blast (Figure 7). The right side of the brain also exhibited no signs of damage by these methods. Moreover, neither astrocytic GFAP immunolabeling nor microglial IBA1 immunolabeling was elevated following any of the blasts. The basal ganglia and amygdala also showed no obvious pathology in sections stained by any of these approaches.

The 50–60-psi blasts did yield axonal degeneration in several brain regions, including the optic tract, pyramidal tract, lateral lemniscus, and the cerebellar peduncles, as revealed by NeuroSilver staining (Figure 7). Thus, although the blast was centered on the cerebral cortex, the brain deformation caused by the blast shock wave caused damage to axons in many major fiber tracts, in that regard resembling the diffuse axonal injury caused by mild TBI in humans. Although it is uncertain if axonal injury underlies the emotional deficits described above, it seems a likely contributor to motor and visual deficits (not detailed here) that we have observed after 50–60 psi blasts.

**Figure 8:**
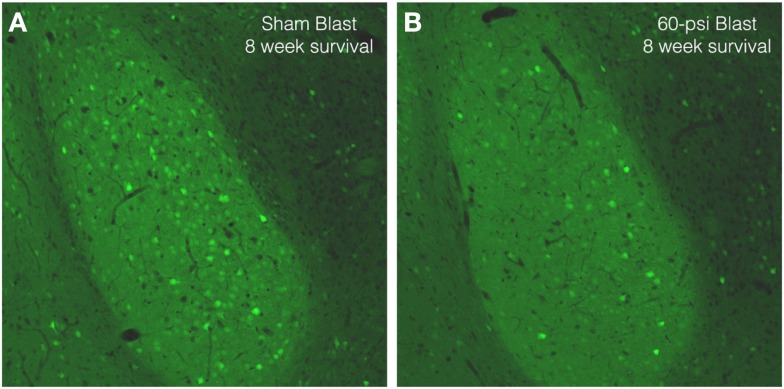
**Images of sections through the basolateral amygdala on the blasted side from mice engineered to express EYFP in Thy1-enriched telencephalic neurons**. Note that the basolateral amygdala (BLA) in the 0-psi mouse **(A)** contains numerous Thy1-EYFP+ neurons, while the BLA in the 60-psi mouse **(B)** at 8 weeks after the blast contains far fewer such neurons.

To further examine brain pathology, we subjected a series of mice conditionally expressing EYFP in Thy1-expressing pyramidal neurons of the telencephalic *emx1* lineage to blast and subsequently examined their brains. In the cerebral cortex, EYFP+ neurons were abundant in layers 3 and 5, with no obvious differences in their abundance or morphology between mice with 50 and 60 psi blasts as compared to 0-psi. In the BLA, however, there were fewer Thy1-EYFP+ neurons in mice after 50–60 psi blasts (Figure 8), with a significant 25.7% reduction for the left BLA and a 10.8% reduction for the right BLA, in a set of nine 0-psi and fifteen 50–60 psi mice with post-blast survivals ranging from 1 to 80 days. Given that Thy1+ BLA neurons have recently been shown to suppress fear retention via a projection to medial central amygdala (9), abnormalities in this neuronal subpopulation may contribute to the increased anxiety and fear in mice subjected to 50–60 psi blast.

## Discussion

In our mouse model of closed-head TBI, we have found that a single 50–60 psi overpressure air blast produces anxiety-like behavior, as evidenced by decreased lingering in the middle of an open field arena, at 2 weeks after blast. This observation led us to more extensively evaluate these mice for emotional abnormalities. We found that in addition to the open field anxiety, 50–60 psi blast mice showed increased acoustic startle (indicative of anxiety), diminished PPI (indicative of neuropsychiatric disorder), increased depression-like behavior, increased contextual fear, and perseverance of cued learned fear over the 2–8-week period after blast. Mice sacrificed after behavioral testing for histological assessments showed no major neuron loss in the brain, although scattered axonal degeneration was observed. Thus, mice with 50–60 psi blast exhibit the minimal neuronal injury coupled to diffuse axonal injury that is characteristic of human mild TBI and is associated with emotional disorders (1–7). Using mice engineered to express EYFP in Thy1+ telencephalic pyramidal neurons, we also found a decrease in EYFP+ neurons in the BLA after 50–60 psi blasts. We do not know if the decrease in EYFP+ BLA neurons stems from an injury to their axons or to their perikarya. We also have not yet determined if the decrease reflects a loss of staining, and perhaps impaired neuronal function, or the actual loss of some EYFP+ BLA neurons. Regardless of the exact scenario, however, the reported link of Thy1+ pyramidal neurons in BLA to fear suppression (9) suggests their damage may contribute to the heightened anxiety and/or fearfulness we observed in mice subjected to 50–60 psi blast. Moreover, this BLA subpopulation appears to be preferentially driven by excitatory input from layer 5 neurons of the medial prefrontal cortex (58). Thus, in principle, neuronal loss or dysfunction of the amygdalofugal neurons of medial prefrontal cortex and/or of the Thy1-enriched BLA neurons could account for the increased fear and anxiety after TBI in our mice. Interestingly, frontal lobe damage is common in human TBI, and frontal lobe damage has been linked to emotional disorders (10–12). Further study of neuronal dysfunction and/or loss in the medial prefrontal cortex and in the Thy1-enriched BLA neurons in our model would aid understanding of their possible contribution to the pathophysiology of the emotional disorder seen in humans with mild TBI.

We also found that single 25–40 psi overpressure air blast directed at the left side of the cranium yielded no obvious brain pathology or motor deficits but produced transient hyperanxiety, as seen in open field at 1 week but not at 2 weeks. In accord with this brief period of emotional impairment, mice subjected to 30-psi blast did not show depression when tested at 2 weeks, or contextual fear when tested at 3 weeks. Thus, the TBI caused by a 25–40-psi blast in our mouse model of closed-head TBI resembles a very mild concussive event in humans that causes only transitory symptoms. Whether 25–40 psi overpressure air blasts in mice would yield more severe and enduring deficits if they were to be repeated is unknown. Such an outcome seems likely, given the evidence from human and animal studies that the effects of a given concussive event are worsened by the occurrence of prior events (59–63), and that a history of subconcussive events can lead to the progressive neurodegenerative condition termed chronic traumatic encephalopathy, with its associated cognitive and emotional deficits (64, 65).

Despite concern about the increasing frequency of mild TBI in humans and its associated emotional disorders, insight into the underlying pathophysiology of such disorders has been lacking. Many animal models of TBI use either fluid percussion impact or controlled cortical impact, both of which involve direct open skull contusive damage to brain that destroys much of the brain parenchyma at the site of impact, and thus causes a far more severe injury than people experience from exposure to air pressure shock waves or from rapid head acceleration–deceleration. Closed-skull controlled impact and whole-animal blast models produce injuries that better resemble mild TBI in humans. Studies employing mild TBI models of this type that have investigated the link between brain trauma on one hand and emotional symptoms on the other are discussed below.

Of the studies that have used a weight drop on a closed skull in rodents, several types of abnormalities have been observed in some studies, but not others. For example, enhanced anxiety in elevated plus maze was seen at 1–2 weeks after weight drop in rats in one study (18), but diminished anxiety was reported at 2 weeks after weight drop in another rat study (66). In mice, no increased anxiety was reported in elevated plus maze at either 1, 2 or 3.5 weeks after weight drop in several studies (22, 23, 67), but was detected at 11 days in another (68). Depression was detected in mice at 7 days using the Porsolt swim test (69), and at 3 and 13 days using the tail suspension test (68, 70). Some studies in mice reported heightened fearfulness in passive avoidance tasks in the month after weight drop TBI (22), while others reported reduced fear learning (69), and others no change (23, 70). In rats, Meyer et al. (18) reported increased learned contextual fear at 1–2 weeks and Pandey et al. (66) reported abnormalities in social behavior at 2 weeks. Motor performance on rotarod or in open field, and learning deficits were observed in most rodent studies (19, 22, 23, 67, 68, 71, 72), but not in all (18, 66). Thus, while motor and cognitive deficits are typically reported, the results concerning anxiety, fear and depression tend to be inconsistent and have been limited to a month after the TBI event. Moreover, although some studies examining the adverse emotional effects of weight drop TBI reported brain pathology (18, 19, 22, 23, 66, 68, 70), none have shown a clear link between the brain pathology and any particular emotional abnormality.

Studies using a shock tube to create mild TBI using blast overpressure in rodents have also tended to produce inconsistent emotional deficits. For example, Koliatsos et al. (17) used a shock tube to produce TBI in mice with single blasts of 25-psi, and evaluated the outcome over a 2-week period after blast. The shock tube blasts produced diffuse axonal injury in the optic tract and brainstem, similar to what we observed. There was a pronounced motor deficit on rotarod at 1 week, but not at 2 weeks, and also transient deficits in social recognition and spatial memory. They, however, found no evidence of enhanced anxiety in an open field arena at either 1 or 2 weeks after injury. Similarly, Kamnaksh et al. (73) used 21-psi overpressure blast to produce mild TBI in rats, and observed no anxiety on elevated plus maze 1 day after the blast. Using similar blast conditions in rats, Kovesdi et al. (20) observed diminished anxiety on elevated plus maze at 44 days after blast, which resolved by 66 days post-blast. Following 5 daily 20-psi overpressure blasts in rats, Kamnaksh et al. (21) observed increased anxiety in open field 1 day after blast, but did not report results beyond 1 day. Elder et al. (16) investigated the long-term effects of TBI in rats exposed to three daily 10.8-psi overpressure air blasts. The blasts produced no overt histopathology, and no changes in motor behavior or in memory tasks were detected. Behavioral tests conducted at 6 months after blast revealed increased open-avoidance in elevated zero maze (indicative of increased anxiety), enhanced acoustic startle, an increased fear response to predator odorant, and increased retention of learned fear to an auditory cue. They did not, however, observe any changes in PPI.

The behavioral changes our mice exhibited after a single 50–60 psi blast were very similar to those exhibited by rats in Elder et al. (16) following three 10.8-psi blasts, except that we observed a clear deficit in PPI, as well as enhanced depression. In our case, we do not know if the deficits we observed at 2–8 weeks, would last 6 months. In the case of Elder et al. (16), the time course over which deficits developed before they were examined at 6 months is not known. Our results do show, however, that a single sufficiently powerful concussive blow to the cranium can produce diverse lasting emotional deficits, and multiple blasts are not needed *per se*. It is uncertain if a single 10.8-psi overpressure air blast using the Elder et al. shock tube would yield the emotional deficits that were seen with three blasts. Repeated concussive episodes may be needed if the individual blast intensities are low. For example, a single 20-psi air blast directed at the cranium using our overpressure blast model did not produce open field anxiety, but multiple 20-psi blasts might. It also may be that a 10.8-psi overpressure air blast to the entire animal is inherently more injurious than a 20-psi blast to the cranium, because the former may also produce lung and vascular damage that exacerbates any brain damage.

The severity of injury, timing of the behavioral test after the injury, and the nature of the test may all be factors contributing to the differences between our results and prior studies using weight drop or shock tube blast to produce TBI (as well as to the inconsistencies among the other studies described above). Importantly, more severe injuries may produce cognitive deficits that result in what may appear to be lessened or normal anxiety on standard anxiety tests such as open field or elevated plus maze (16, 21, 23, 68, 70, 73). The use of several tests of anxiety (open field and startle) and fear (fear acquisition, fear extinction, and learned contextual fear), as in the studies we report here, as well as tests of depression (tail suspension) and possible neuropsychiatric disorder (PPI) provide a better means of evaluating the effects of mild TBI on emotional disorders.

In summary, we found that a single 50–60 psi air pressure blast to the left mid cranium in C57BL/6 mice leads to emotional abnormalities that persist for at least 8 weeks after blast. These abnormalities included enhanced open field anxiety, increased auditory startle, heightened depression, increased retention of learned fear to an auditory cue, and increased contextual fear after fear learning, as well as a deficit in PPI. Our results thus show that single air pressure blasts that yield no prominent neuronal loss in the forebrain are sufficient to produce a variety of emotional deficits similar to those seen in human victims of mild TBI (1–3, 5–7). Although the precise brain damage responsible for the deficits we saw remains to be established, our data suggest that the fear and anxiety deficits, at least, may be related to dysfunction or loss of Thy1-enriched neurons of the BLA. Overall, our model of a single air overpressure blast to the cranium to produce mild TBI without systemic, whole-body effects provides a new model for controlled investigation of the consequences of TBI without confounding secondary effects of the blast on the lungs or the cardiovascular system.

## Author Contributions

Primary Roles: Scott A. Heldt – research, analysis, manuscript preparation; Andrea J. Elberger – research, analysis, manuscript preparation, grant support; Yunping Deng, Natalie H. Guley, Nobel Del Mar, Joshua Rogers, Gy Won Choi, Jessica Ferrell – research, analysis; Tonia S. Rex – research, grant support; Marcia G. Honig – research, analysis, manuscript preparation; and Anton Reiner – research, analysis, manuscript preparation, grant support.

## Conflict of Interest Statement

The views, opinions and/or findings contained in this research presentation are those of the authors and do not necessarily reflect the views of the Department of Defense and should not be construed as an official DoD/Army position, policy or decision unless so designated by other documentation. No official endorsement should be made.
